# Enhanced mechanical properties and environmental stability of polymer-bonded magnets using three-step surface wet chemical modifications of Nd–Fe–B magnetic powder

**DOI:** 10.1016/j.heliyon.2024.e26024

**Published:** 2024-02-11

**Authors:** Ana Damnjanović, Ingrid Milošev, Nataša Kovačević

**Affiliations:** a Kolektor Mobility d.o.o, Vojkova Ulica 10, SI-5280, Idrija, Slovenia; b Jožef Stefan International Postgraduate School, Doctoral Study Programme Nanoscience and Nanotechnologies, Jamova C. 39, SI-1000, Ljubljana, Slovenia; c Jožef Stefan Institute, Department of Physical and Organic Chemistry, Jamova C. 39, SI-1000, Ljubljana, Slovenia

**Keywords:** Nd–Fe–B, Tetraethyl orthosilicate (TEOS), γ-aminopropyltriethoxysilane (APTES), Surface modification, Permanent magnets, Corrosion resistance

## Abstract

This research focuses on the surface modification of Nd–Fe–B magnetic powder to enhance its thermal and oxidation resistance without compromising magnetic properties and to improve adhesion to the polymer binder for enhanced mechanical properties. A three-step surface modification process involving phosphatization treatment, tetraethyl orthosilicate (TEOS) application, and 3-aminopropyltriethoxysilane (APTES) grafting, was applied to the powder, which was then compounded with polyamide 12 and injection-moulded into cylinders and dog-bone-shaped tubes. The resulting magnets exhibited remanence (B_r_) of 487.6 mT, coercivity (H_ci_) of 727.7 kA/m, and energy product (BH_max_) of 39.3 kJ/m^3^. The modified magnets demonstrated exceptional corrosion resistance and thermal stability, with less than 5% irreversible flux loss after exposure to hot water, temperature shock, and pressurised steam. Furthermore, the modified magnets displayed significantly higher tensile strength, elongation at break, and elastic modulus with improvements of 62%, 16.7%, and 19.9%, respectively, compared to the non-modified batch. Additionally, the modified batch showed a notable 52% increase in flexural stress during flexural testing. These findings underscore the potential of silane surface modifications in producing injection-moulded permanent magnets based on Nd–Fe–B alloy, extending their shelf life and enhancing their overall performance.

## Highlights

• Nd-Fe-B powder was treated using three-step wet chemical surface modification.
• Improved interaction between Nd-Fe-B and polyamide 12 enhances mechanical properties.
• Bonded magnets with 62% higher tensile strength and 52 % higher flexural strength.
• Improved corrosion resistance and thermal stability with minimal flux loss.

## Graphical abstract

**Figure:**
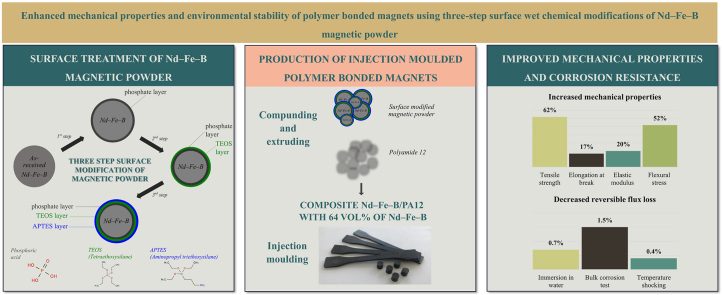

## 1 Introduction

In recent decades, polymer-bonded magnets (PBMs) based on Nd–Fe–B alloys have emerged as highly promising materials with significant contributions to technological advancements. They offer higher energy products in reduced volumes than conventional materials, making them indispensable in modern technologies. These magnets are crucial in ‘green technology’ applications such as electric vehicles and wind turbines, which are vital for addressing climate change concerns [1].

The manufacturing of PBMs involves mixing magnetic powder with a binder and using either compression or injection moulding. Recently, additive manufacturing methods like fused deposition modelling have also been utilised for fabricating PBMs [2,3]. Injection moulding, although requiring more polymer for better flow (35–40 vol%), allows for complex shapes but results in slightly reduced magnetic strength due to higher binder content [4,5]. PBMs offer greater design flexibility compared to sintered or hot-deformed types and can be used in a variety of applications, from computer storage to automotive products.

Depending on desired properties and final applications, various polymers can be utilised as binders. The commonly employed binder is solid epoxy resin, owing to its convenient moulding and processing characteristics in the industrial production of bonded magnets. Nevertheless, the use of bonded magnets in advanced industries has been hindered by the limited ability of epoxy binders to withstand high temperatures, resist corrosion, and maintain satisfactory mechanical performance [6]. Regarding moldability and mechanical strength, binder resins such as thermoplastic polyamide 12 (PA12) are preferable because they allow for higher filler loadings of up to 70 vol% [[7], [8], [9]]. Other polymers, such as polyphenylene sulfide, offer advantages in terms of heat resistance but require a smaller filler load [10]. Polyether Ether Ketone, utilised in space applications, complies with stringent aerospace requirements by having high thermal stability, low outgassing, low flammability, and high radiation resistance [11]. By selecting an appropriate binder polymer based on desired properties and applications, enhanced performance and expanded possibilities for bonded magnets can be achieved.

However, there is a pressing need for more sustainable usage of PBMs, given their susceptibility to oxidation and corrosion, which can shorten the lifespan of magnet assemblies. One approach to enhance sustainability involves depositing functional coatings on magnetic substrates. These coatings improve surface properties, including oxidation resistance, mechanical strength, and electrical and thermal conductivity [12]. Another strategy focuses on the compatibility between magnetic particles and polymers. To improve this interaction, various methods have been studied, and silane coupling agents have emerged as particularly effective. The silanes serve dual purposes: they facilitate the mechanical bonding of magnetic powder and polymer and maintain desirable magnetic properties and corrosion resistance.

Surface modification methods employing organo-silane coupling agents have attracted significant attention because of their remarkable corrosion resistance and strong adhesion properties. The primary chains of organic polymers, characterised by siloxane bonds (Si–O–Si), possess higher bond energies than C–C and C–O bonds. The helical structure and presence of organic groups within these bonds contribute to reduced intermolecular forces, leading to decreased surface tension and enhanced water repellence. Coating agents with the general formula R–Si−(OR')_n_, where R and R′ represent alkyl groups, and *n* is either 3 or 4, exhibit promising potential [13]. Applying a monolayer of the coating agent onto the surface of the powder offers several advantages. First, it reduces the viscosity of suspensions, resulting in improved dispersion. Second, it enhances the hydrophobicity of the powder surface, leading to enhanced water repellence. Finally, it facilitates compatibility with the polymer matrix by enabling chemical bonding between the powder and the polymer through hydrogen or ionic bonds. These interactions strengthen the overall adhesion between the coated powder and polymer matrix, thereby enhancing the performance of the composite material [8,14,15]. Applying a silane coupling agent to coat Nd–Fe–B powders offers the necessary resistance to oxidation and corrosion during processing. Various silane agents can be utilised to treat the surface of Nd–Fe–B, such as tetraethoxysilane [16], *N*-(2-aminoethyl)-3-aminopropyltrimethoxysilane [17], 3-aminopropyltriethoxysilane [18] and (3-glycidoxypropyl)trimethoxysilane [19]. These silane agents play a crucial role in enhancing the stability and protective properties of the surface of Nd–Fe–B powders, ensuring their suitability under the intended processing conditions. In commercial applications, silanes are commonly mixed with magnetic powder in liquid form to act as coupling agents. However, ensuring a homogeneous mixture throughout the magnetic powder can be challenging. This study aims to evaluate a three-step surface modification of Nd–Fe–B magnetic powder for its impact on the mechanical properties and environmental stability of injection-moulded PBMs. Additionally, the variability in the volume ratio of polymer to magnetic filler, as well as the use of diverse polymer, fundamentally changes the mechanical properties, affects the environmental stability and magnetic characteristics of these magnets. Therefore, to maintain the integrity and specificity of our findings, we have consciously chosen not to use the work of other authors for comparison in this study.

## 2 Materials and methods

### 2.1 Materials

The magnetic filler used in this study was a commercial isotropic MQP B+ (−150 mesh) powder provided by Magnequench. MQP B+ powder, composed of an Nd–Fe–Co–B alloy, was produced using a melt-spinning process, forming particles with an irregular flake-like morphology. As-received magnetic powder exhibited the following magnetic properties: residual remanence (B_r_) within the range of 895–915 mT, coercivity (H_ci_) between 716 and 836 kA/m, and a maximum energy product (BH_max_) of 126–134 kJ/m^3^ [20]. Polyamide 12, a polymer binder, was used in either powder or pellet form (Vestosint and Vestamid, respectively, from Evonik, Italy). A phosphoric acid solution was employed as a first step, while tetraethyl orthosilicate (TEOS) was utilised as a coupling agent. APTES (3-aminopropyltriethoxysilane) was grafted onto the TEOS layer as the final modification step. Isopropyl alcohol (IPA) was used as a dilution solution. All chemicals were supplied by Sigma-Aldrich (Slovenia).

### 2.2 Surface modification of Nd–Fe–B powder

Magnetic powder particles were coated using patented technology/procedure [21]. The powder coating was performed using a Reactor-Ready pilot lab reactor (Radleys, UK), which comprised a reaction vessel, an overhead PTFE anchor stirrer, and a vacuum-jacketed option connected to a Huber Ministat 230 thermoregulator/circulator. The integration of these components enables rapid heating or cooling within the system, which is essential for producing high-quality powder coatings.

The first step involved a phosphating treatment, wherein a mixed solution of 0.5 wt% phosphoric acid solution, 2.5 wt% IPA and MQP B+ were added to the lab reactor and stirred for 10 min in air at atmospheric pressure. The mixture was then heat-treated for 1 h at 80 °C and subsequently for 2.5 h at 120 °C. However, because the volume of the solution was insufficient for a uniform dispersion, more IPA was added.

In the second step, a composite coating layer is obtained by adding a mixed solution of 0.7 wt% TEOS, 0.30 wt% phosphoric acid, 0.26 wt% deionised water (DI), and 2.5 wt% IPA to the powder obtained in the previous step and mixed for 10 min. Because the volume of the diluting solution seemed insufficient for a uniform dispersion, more IPA was added. The obtained mixture was heat-treated for 1 h at 80 °C and subsequently for 2.5 h at 120 °C in air at atmospheric pressure while stirring.

In the final step, MQP B+ powder coated with a phosphate layer and a TEOS layer from the first and second steps was subject to the silane coupling agent APTES, which exhibits the best rust-prevention properties. The coated powder was mixed with a solution of 0.5 wt% APTES, 2.5 wt% IPA, and 0.3 wt% DI, stirred for 10 min in an air atmosphere and then heat-treated while stirring at 100 °C for 1 h in the air at atmospheric pressure.

All powders obtained were cured in an oven at 100 °C for 1 h.

**Fig. 1:**
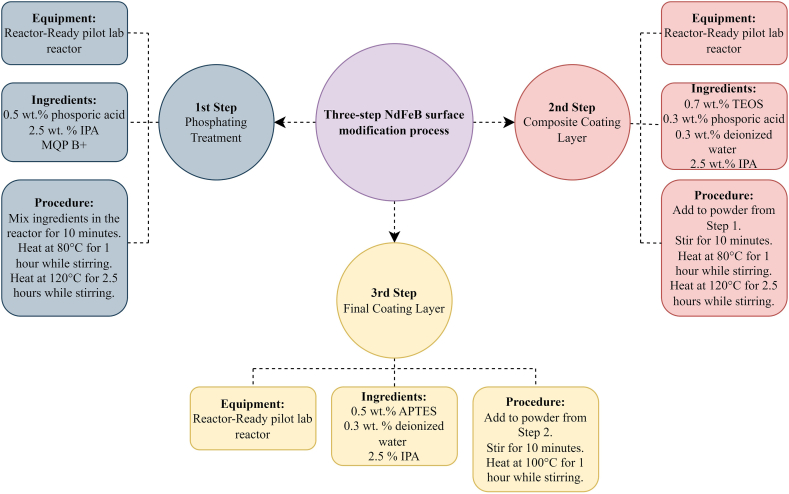
Schematic representation of the three-step surface modification process of the batch MQP B+/H_3_PO_4_/TEOS/APTES.

Several batches were produced to evaluate the differences that may arise between the presence and absence of TEOS and APTES, respectively, as presented in Table 1. The surface modification of the batch, subsequently employed in the industrial manufacturing of PBMs, followed a three-step process, as illustrated in Fig. 1.

**Table 1:** Overview of magnetic powder batches with different coating layers.

Batch name of magnetic powders	Surface modification
As-received MQP B+	none
MQP B+/H_3_PO_4_	phosphate coating
MQP B+/H_3_PO_4_/TEOS	phosphate + TEOS coatings
MQP B+/H_3_PO_4_/TEOS/APTES	phosphate + TEOS + APTES coatings
MQP B+/H_3_PO_4_/APTES	phosphate + APTES coatings

### 2.3 Injection moulding of Nd–Fe–B bonded magnets

PBMs were produced in manufacturing facilities at Kolektor KFH. Before injection moulding, the magnetic powder was mixed with additives by combining 99 wt% of filler and 1 wt% of additives for 2 min. Additives were added to provide a better dispersion of the polymer, better flow properties, and less friction. This mixture was added to the first dosage unit of the two-screw extruder in a 93 wt%, and in the second dosage unit, 7 wt% of PA12. The temperature in the multibarrel zone extruder ranged from 160 °C to 230 °C. The resulting mixture was kneaded, extruded, and cut into pellets.

Subsequently, the pellets were injection-moulded to produce the bonded magnets. The temperature range in the injection moulding unit ranged from 230 °C to 295 °C, with the pressure varying from 804 to 868 bar. PBMs were produced in the shape of cylinders and dog-bone-shaped tubes. The total length of the test tube was 150 mm, with a 50 mm– test section gauge length and 20 mm wide, whereas the injection-moulded cylinders were 10 mm × 9.9 mm. Dog-bone-shaped tubes were produced to evaluate mechanical properties, whereas cylinders were to evaluate environmental stability.

Two batches were produced. The first batch included as-received MQP B+ powder and PA12. Cylinders were made of as-received MQP B+ powder and PA12 powder, whereas dog-bone-shaped-tubes from as-received MQP B+ powder and PA12 pellets. This is because the manufacturing process was optimized for PA12 pellets, and only cylinders were injection-moulded without defects with PA12 powder. Therefore, we used dog-bone-shaped tubes from KFH regular production, i.e., as-received MQP B+ powder bonded with PA12 pellets. The second batch included MQP B+ powder, modified with phosphate and subsequently with silanes, bonded with powdered PA12, and labelled modified MQP B+/PA12.

### 2.4 Characterisation

Different methods were used to characterise the surface of the as-received MQP B+ powder and modified powder from batch MQP B+/H_3_PO_4_/TEOS/APTES and evaluate the efficiency of the coating layers against corrosion. Fourier-transform infrared spectroscopy (FTIR) was used to obtain the infrared spectrum of absorption of the as-received and coated powders. FTIR spectra of samples were measured by PerkinElmer Spectrum 100, using an attenuated total reflectance mode ATR sampling accessory.

Magnetic measurements of the uncoated and coated powders were performed using a vibrating sample magnetometer (VSM, LakeShore 7307) at room temperature. All samples were first magnetized before the VSM measurement. For this measurement, hysteresis loops were plotted as magnetization, M as a function of the applied field, and H (M − H loop).

The as-received and modified powders were subjected to thermogravimetric analysis (TGA) to determine the effect of temperature on the oxidation and corrosion susceptibility. TGA tests were performed on the uncoated and coated samples using a Mettler Toledo thermogravimetric analyser TGA/DSC1. The test conditions were the same for all samples in the non-isothermal mode. The samples were heated from ambient temperature to 600 °C at a heating rate of 10 °C/min in the presence of air as the purge gas.

The surface morphology and chemical composition of the as-received and modified, and the injection-moulded magnets, were determined using scanning electron microscopy (SEM) coupled with energy-dispersive X-ray analysis (EDX). SEM images and EDS spectra were taken in secondary electron mode using Jeol–IT300 SEM equipped with Oxford EDX at 15 keV energy beam (JEOL ltd). Samples were not pretreated; they were fixed on a carbon tape before analysis.

Injection-moulded cylinders were magnetized using an impulse magnetizer K-Series (MAGNET-PHYSIK) at a voltage of 2000 V to saturate the samples. After magnetization, residual remanence (B_r_) and intrinsic coercivity (H_ci_) were measured using a permeameter (PERMAGRAPH®, MAGNET-PHYSIK).

The magnetic flux of the magnetized cylinders was measured using a Helmholtz coil (MS 75 with electronic Fluxmeter EF 14, MAGNET-172 PHYSIK), both before and after subjecting them to environmental tests.

Mechanical testing was performed on five dog-bone-shaped specimens at room temperature. The test specimens were injection-moulded in specified dimensions according to the standard ISO 527 for 1B type test specimens. Mechanical tests consisted of flexural and tensile tests. The flexural test was conducted using a Zwick test machine (Z100) following standard ISO 178:2001 at a test speed of 1 mm/min [22]. Tensile tests were performed on the same test machine as a flexural test, with different setups, following ISO 527 [23]. The testing speed was 1 mm/min with a load cell of 100 kN.

### 2.5 Evaluation of environmental stability and corrosion resistance

The corrosion resistance of Nd–Fe–B permanent magnets in humid and chloride-containing environments was reported to be remarkably low [24], which is attributed to the reaction of the Nd-rich phase with H_2_O, resulting in the formation of Nd(OH)_3_ and causing decohesion and dropout of magnetic grains [25]. Such functionality deterioration due to corrosion undermines the strength of the magnets, leading to irreversible loss in flux and, in severe cases, complete disintegration of the magnet.

In this study, the environmental stability and corrosion resistance of Nd-Fe-B magnets were evaluated by measuring the flux loss after exposure to four test environments. The percentage of irreversible flux loss after the tests, after the remagnetization of samples, was calculated to assess environmental stability and corrosion resistance.

To examine the stability of the magnets in an aqueous environment, the magnetized cylinders were immersed in deionized (DI) water at 120 °C and corrosive water at 95 °C for 2000 h, respectively. The corrosive water immersion test was performed following standard ASTM D1384 [26]. The loss of magnetic flux was measured at seven-day intervals after the immersion processes.

For the Bulk Corrosion Test (BCT), the cylinders were subjected to pressurized steam at 120 °C for 96 h to assess the material degradation under the combined effect of heat and water vapour.

To simulate the effects of rapid temperature changes that may occur during the use of Nd-Fe-B magnets, cylinders underwent temperature shock in air, involving 900 cycles of exposure to temperatures ranging from −40 °C to +140 °C for 30 min each, with a chamber transfer time of less than 10 s.

All the above-mentioned tests were performed on at least three magnetized cylinders for each batch.

## 3 Results and discussion

### 3.1 Characterisation of as-received and modified magnetic powder

In the referenced literature, an alkoxy oligomer capped with an alkoxysilyl group is recommended for the coating treatment. Among the alkoxy groups, ethoxy groups are preferred due to their reactivity and compatibility, which is why we selected tetraethyl orthosilicate (TEOS), which is a widely accepted choice in materials science for such applications because of its ethoxy groups [21].

Additionally, for the final coating treatment, we selected APTES (3-amino propyltriethoxysilane) from the recommended list of silane coating agents. APTES is renowned for its ability to enhance the interface between polyamide matrices and inorganic materials through its reactive amino and ethoxysilyl groups. These groups facilitate the formation of silanol groups that can bond with substrate surfaces, thus improving filler dispersion and interfacial adhesion thus contributing to the mechanical robustness and stability of the composites [27].

**Fig. 2:**
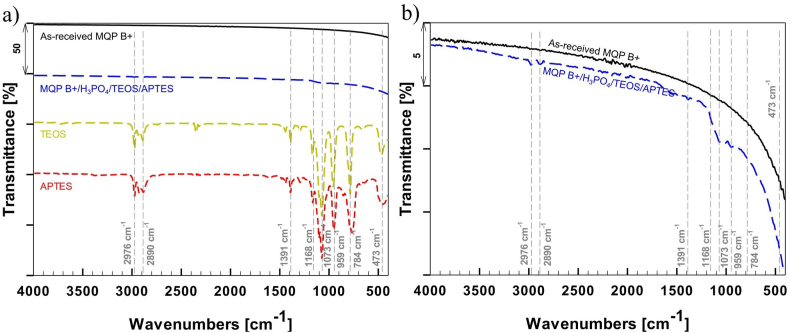
FTIR spectra comparison of as-received MQP B+ powder, modified MQP B+ powder, and liquid silanes TEOS and APTES, with a transmission scale up to 50% (a) and FTIR spectra of as-received MQP B+ powder and modified MQP B+ powder with transmission scale up to 5% (b).

Fig. 2 compares the FTIR spectra, depicting the as-received and modified MQP B+ powders and TEOS and APTES. The modified powder exhibits a peak at approximately 1100 cm^−1^, indicating the presence of Si–O–Si and Si–O–C bonds originating from APTES on the powder surface (Fig. 2b). Moreover, an additional characteristic peak corresponding to C–H stretching from COC was observed at 2900 cm^−1^ [28]. The spectral peaks observed on the surface of the modified magnetic powder are in line with those derived from TEOS and APTES silanes (Fig. 2a). This evidence supports the presence of silane groups on the surface of the modified magnetic powder, thereby confirming the successful implementation of the silane layer.

**Fig. 3:**
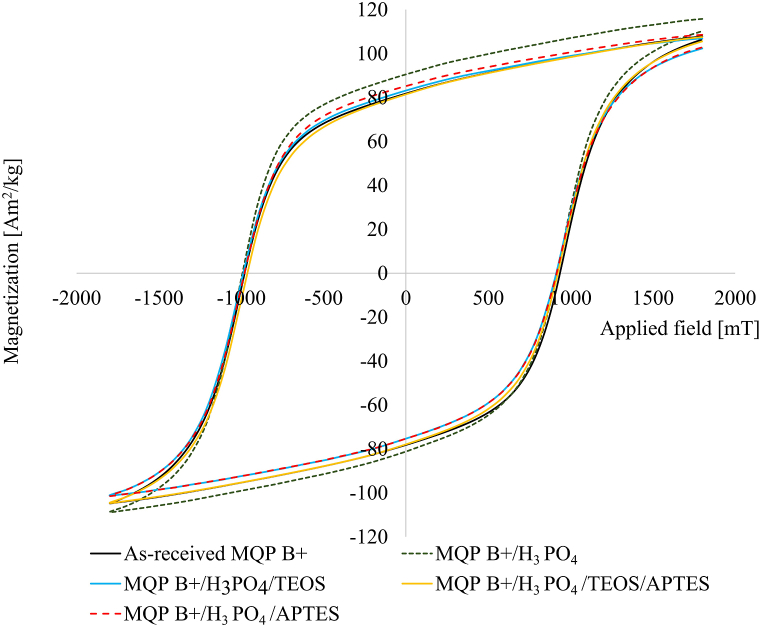
Hysteresis curves obtained via VSM measurements for the as-received and different batches of modified MQP B+ powder.

The results of the VSM measurements, as presented in Fig. 3, demonstrated that the intrinsic magnetic properties of the modified powders remained unaffected by the surface coating process. Specifically, the modified specimens exhibited magnetic characteristics similar to those of the as-received powder. This observation was particularly notable for the samples that underwent a three-step modification process, initially with phosphate and subsequently with silanes, as their hysteresis loops overlapped with those of the uncoated powder, confirming the preservation of their intrinsic magnetic properties.

**Fig. 4:**
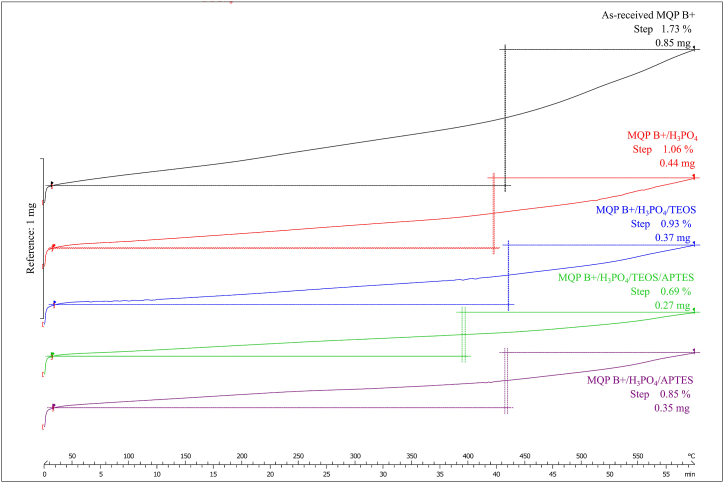
TGA curves illustrate weight gain (y-axis) over time with increasing temperature (x-axis) for different batches of MQP B+ powder. The bar representes a scale reference for y-axis.

The advantageous effects of applying the powder coating are depicted in Fig. 4, which shows the TGA curves. It is evident that the initial weight gain began simultaneously for all samples and increased with temperature over time. The observed increase in the weight can be attributed to the formation of oxidation products during the measurement process. Notably, powders with distinct coating layers exhibited diverse behaviours. The TGA curve demonstrated the highest weight gain of 1.7% for the as-received powder. Conversely, the MQP B+/H_3_PO_4_/TEOS/APTES sample, where the powder was phosphatized to improve the adhesion of silane layers, followed by TEOS coating and subsequent grafting of APTES, exhibited the lowest weight gain of 0.7%. These findings suggest that this specific composite coating offers superior thermal stability for the Nd–Fe–B powders. Consequently, it was selected as the feedstock material for industrial-scale injection moulding, and the moulded samples were subsequently subjected to further evaluation.

**Fig. 5:**
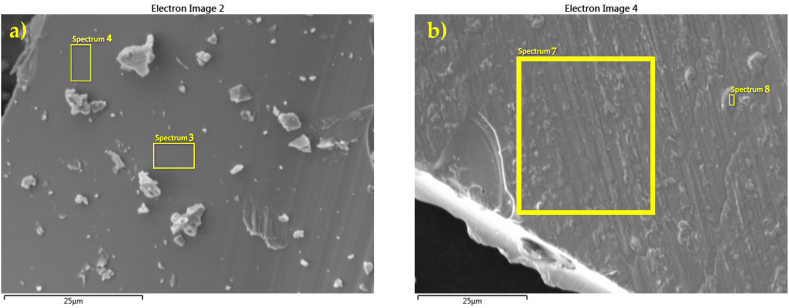
SEM images comparing as-received MQP B+ (a) and modified MQP B+ (b) powder. Areas where EDS analysis was conducted are denoted by enumerated rectangles; the results are given in Table 2.

The morphologies and compositions of the as-received and modified MQP B+ powders were investigated using electron microscopy with chemical analysis (SEM-EDX). The SEM micrographs presented in Fig. 5 highlight the effects of the melt-spun process, which resulted in the formation of irregularly shaped flake-like particles with noticeable melting traces and an uneven surface. The melt-spun process is followed by crushing, leading to the blocky morphology observed in the MQP B+ powder. To assess the elemental composition of the powders, the as-received samples, and the modified MQP B+ powder were compared. As indicated by the elemental distribution in Table 2, there is a minor presence of P, N, and Si on the surface of the modified MQP B+ powder, originating from the coating layers.

**Table 2:** The elemental compositions (wt. %) of the as-received MQP B+ powder and modified MQP B+ powder were determined by EDX spectrum analysis (SEM images in Fig. 5).

Spectrum label	As-received MQP B+ powder		modified MQP B+ powder	
	Sp. 3	Sp. 4	Sp. 7	Sp. 8
Nd	28.2	28.0	28.0	27.4
Fe	65.7	65.5	63.5	63.9
Co	5.5	5.5	5.5	5.4
O	1.0	1.0	2.0	2.3
P	/	/	0.3	0.3
Si	/	/	0.5	0.6
N	/	/	0.2	0.1
Total	100.0	100.0	100.0	100.0

### 3.2 Injection-moulded magnets characterisation

The results of the magnetic measurements are summarized in Table 3. The intrinsic characteristics of the material, such as the coercivity (H_ci_), are influenced by the composition of the initial material. The obtained H_ci_ values for injection-moulded cylinders align with those of the as-received powder. For the 65% volumetric loading of the magnetic filler utilised in our study, the expected remanence value was 585 mT. The injection-moulded cylinders achieved 83% of their theoretical B_r_ values. However, it should be noted that polymer binders are typically used in the form of pellets during the manufacture of magnets in an industrial setting. The lower B_r_ values observed in this study may be attributed to the suboptimal parameters employed for injection moulding using powdered polymer binder. The coating layers did not diminish the magnetic properties, as evidenced by the VSM hysteresis analysis of the modified and as-received powders (Fig. 3). The remanence (B_r_) and energy product (BH_max_) remained similar for both the modified and non-modified batches, providing further confirmation from previous VSM measurements.

**Table 3:** Measured magnetic properties of injection-moulded samples (with a minimum of three samples per measurement).

Batch	B_r_ [mT]	H_ci_ [kA/m]	BH_max_ [kJ/m^3^]
As received MQP B+ powder	895–915	716–836	126–134
MQP B+/PA12	486 ± 1.3	719 ± 2	39.3 ± 0.2
modified MQP B+/PA12	487.6 ± 0.8	727.7 ± 2	39.3 ± 0.2

Furthermore, as mentioned earlier, test dog-bone-shaped tubes produced from the MQP B+/PA12 batch exhibited defects and were unsuitable for mechanical testing. Owing to the lack of optimization in the injection moulding process, the production of defect-free test dog-bone-shaped tubes from the unmodified material failed. Conversely, the successful manufacturing of such tubes was achieved with the modified material, supporting the notion that the surface treatment of powders enhances powder fluidity by reducing the friction between the magnetic powder and the polymer, which is aligned with the previous study [29].

**Fig. 6:**
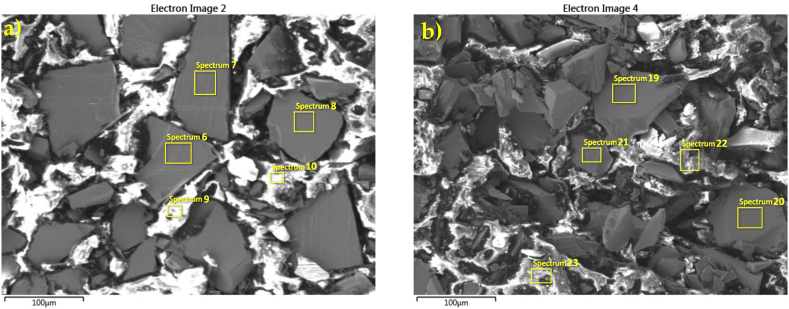
SEM images of the injection-moulded cylinders produced from as-received (a) and modified (b) MQP B+ powder, both bonded with PA12. Areas where EDS analysis was conducted are denoted by enumerated rectangles; the results are given in Table 4, Table 5

The surface characteristics of the injection moulded cylinders from both batches are depicted in Fig. 6, revealing no apparent contrast between the two. Notably, the homogeneity and distribution of magnetic particles within the polymer matrix were remarkably similar. The elemental compositions (wt.%) of the major elements detected on the surfaces of the magnetic powders and polymer in the cylinders are presented in Table 4, Table 5. In Table 5, the absence of Si and P on the magnetic component surface, compared to their presence on the polymer surface in the polymer matrix, suggests the incorporation of some coating material into the polymer matrix throughout the moulding process. The concentrations of Nd and Fe in the magnetic portion of the cylinders in both batches were comparable. A higher concentration of C suggests that applying the coupling agent TEOS and the subsequent grafting of APTES onto the TEOS layer improved the adhesion between the modified magnetic powder and the polymer matrix. As a result, the higher carbon concentration primarily originates from the polymer matrix that encapsulates Nd–Fe–B particles.

**Table 4:** The elemental compositions (wt.%) of the injection-moulded (IM) cylinders produced from as-received MQP B+ powder, bonded with PA12 (SEM image in Fig. 6a).

Spectrum label	MQP B+ powder in IM cylinder			PA12 matrix	
	Sp. 6	Sp. 7	Sp. 8	Sp. 9	Sp. 10
C	4.5	5.0	4.7	18.4	72.8
O	1.2	1.5	1.4	2.5	4.4
Fe	62.2	61.8	62.1	51.6	14.8
Co	5.0	5.2	5.1	4.3	1.1
Nd	27.1	26.5	26.8	23.2	6.9
Total	100.0	100.0	100.0	100.0	100.0

**Table 5:** The elemental compositions (wt.%) of the injection-moulded (IM) cylinders produced from modified MQP B+ powder bonded with PA12. (SEM image in Fig. 6b).

Spectrum label	Modified MQP B+ powder in IM cylinder			PA12 matrix	
	Sp. 19	Sp. 20	Sp. 21	Sp. 22	Sp. 23
C	7.5	7.1	9.5	68.9	72.6
O	2.6	2.3	2.1	5.1	8.9
Si	/	/	/	0.7	0.5
P	/	/	/	1.0	0.6
Fe	58.8	59.6	58.3	15.8	11.9
Co	5.0	5.1	4.7	1.1	/
Nd	26.0	26.0	25.4	7.6	5.6
Total	100.0	100.0	100.0	100.0	100.0

### 3.3 Mechanical properties

**Fig. 7:**
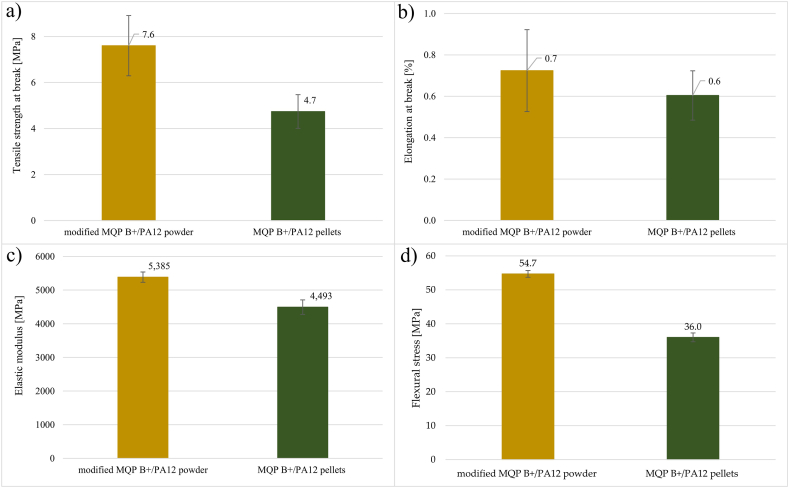
Results of tensile testing for modified MQP B+/PA12 powder and MQP B+/PA12 batches: (a) Tensile strength at break (MPa), (b) Elongation at break (%), (c) Elastic modulus (MPa), and (d) Flexural testing.

The obtained mechanical test results, as depicted in Fig. 7, clearly indicate the effectiveness of the silane coating layers in improving the mechanical properties of the injection-moulded dog-bone-shaped tubes. Specifically, the modified MQP B+/PA12 batch exhibited significantly higher tensile strength, elongation at break, and elastic modulus, with improvements of 62%, 16.7%, and 19.9%, respectively, when compared to the MQP B+/PA12 batch. Moreover, the modified batch also demonstrated a noteworthy 51.9% increase in flexural stress during flexural testing. These results validate the successful enhancement of molecular interaction between the inorganic component (NdFeB) and the organic component (PA12) through three-step modification.

### 3.4 Environmental stability and corrosion resistance

**Fig. 8:**
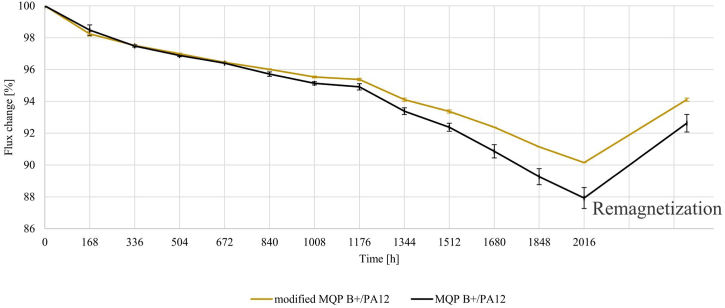
Flux loss of injection-moulded cylinders versus time exposure to corrosive water at 95 °C for 2000 h and after final remagnetization.

**Fig. 9:**
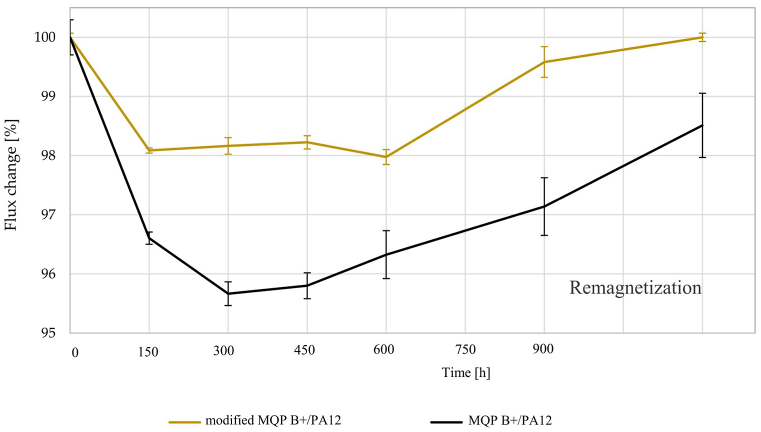
Flux loss of injection-moulded cylinders versus time exposure to temperature shocking on air, to temperatures ranging from −40 °C to +140 °C, for a total of 900 h.

Flux loss in magnetic materials can be divided into reversible, recoverable irreversible, and structural loss. Reversible flux loss, highly dependent on temperature, can be reverted by cooling the magnet, while irreversible flux loss involves persistent changes that do not revert to their initial state even after removing the perturbing factor. The reversible and irreversible flux losses were assessed on injection-moulded cylinders of modified MQP B+/PA12 and MQP B+/PA12 batches. These cylinders were exposed to four distinct scenarios; the results are presented in Table 6. The flux loss over 2000 h in this corrosive environment is depicted in Fig. 8. Fig. 9 provides a graphical representation of the flux loss as a function of time exposure to temperature shocking in air over 900 h. The observed slight increase in flux loss after 300 h aligns with the ± 1% precision of the Helmholtz coil measurement, underscoring the material's thermal stability (Fig. 9). This change confirms the material's capacity to sustain a stable magnetic domain arrangement even under sustained thermal stress. The conditions of water immersion, BCT, and temperature shocking resulted in acceptable flux loss values for both batches. Interestingly, the batch of modified MQP B+/PA12 showed no irreversible flux loss in all three tests, while the MQP B+/PA12 batch showed minor irreversible flux loss of 0.5% in water immersion and 1.5% in temperature shock. This suggests that injection-moulded magnets made from MQP B+ bonded with PA12 are well-suited for applications that involve water immersion or diverse temperature conditions in dry air. Additionally, utilizing modified powder can extend the magnet's lifespan due to reduced flux loss. This correlates with TGA results, which reveal better oxidation resistance for modified powder than as received MQP B+.

**Table 6:** Overview of different environmental stability tests to which modified MQP B+/PA12 and MQP B+/PA12 batches were exposed.

Test Name	Test Temperature/Duration	Sample Name	Reversible Flux Loss [%]	Irreversible Flux Loss [%]	Rusting
Immersion in water	120 °C/2000 h	modified MQP B+/PA12	0.7	0	Low
		MQP B+/PA12	2.9	0.5	Low
Immersion in corrosive water	95 °C/2000 h	modified MQP B+/PA12	9.8	5.9	Severe
		MQP B+/PA12	12.1	7.4	Severe
Bulk corrosion test	120 °C/96 h	modified MQP B+/PA12	1.5	0	Severe
		MQP B+/PA12	2.0	0	Severe
Temperature shocking	−40/140 °C/900 h	modified MQP B+/PA12	0.4	0	Low
		MQP B+/PA12	2.9	1.5	Low

However, a significant flux loss was noted in the test scenario involving immersion of the cylinders in corrosive water at 95 °C for 2000 h. Both batches exceeded the 5% flux loss limit, indicating the unsuitability of these magnets for applications involving aqueous media containing salts. A significant highlight is that the batch made from modified MQP B+ showed lower flux loss than that made from as-received powder. This indicates that the anti-oxidation property can be improved by treating the magnetic powder with phosphoric acid before applying silane treatment using TEOS and subsequent grafting of APTES.

## 4 Conclusions

The main objective of this study was to evaluate the three-step surface modification on magnetic powders and the mechanical properties and corrosion resistance of polymer-bonded magnets. The FTIR and SEM-EDX analyses confirmed the successful coverage of the silane coating on the surface of the modified MQP B+ powder, as indicated by the presence of stretching vibrations associated with Si–O–Si and Si–O–C bonds, and the detection of Si, N, and P elements. Furthermore, VSM measurements demonstrated that the presence of the coating layer did not diminish the magnetic properties of the modified powder.

The modified bonded magnets exhibited lower irreversible flux loss than the non-treated batch in various environments, including water immersion at higher temperatures, exposure to pressurized steam during the BTC test, and temperature shock. However, a significant flux loss was observed when the cylinders were immersed in corrosive water, exceeding the 5% flux loss limit for both batches. These findings suggest that applying a composite silane coating to MQP B+ powder can result in minimal or reduced flux loss in applications involving water immersion or diverse temperature conditions in dry air, compared to non-treated MQP B+ magnets.

Regarding mechanical properties, the flexural and tensile test measurements demonstrated that the presence of the silane coating significantly increased the flexural and tensile strength of the modified bonded specimens.

In summary, applying a silane coating on the magnetic filler successfully enhanced the adhesion between the filler and polymer in the polymer matrix and improved corrosion resistance. The modified bonded magnets demonstrated lower irreversible flux loss and enhanced mechanical properties compared to the non-treated material.

## Funding

This research was funded by the European Union's Horizon 2020 Research and Innovation Program under grant agreement no. 766007.

## Data availability statement

Data will be made available on request.

## CRediT authorship contribution statement

**Ana Damnjanović:** Writing – original draft, Validation, Methodology, Investigation, Formal analysis, Conceptualization. **Ingrid Milošev:** Writing – review & editing. **Nataša Kovačević:** Writing – review & editing, Supervision, Project administration, Conceptualization.

## Declaration of competing interest

The authors declare that they have no known competing financial interests or personal relationships that could have appeared to influence the work reported in this paper.
